# Exposure to fluoridated water and attention deficit hyperactivity disorder prevalence among children and adolescents in the United States: an ecological association

**DOI:** 10.1186/s12940-015-0003-1

**Published:** 2015-02-27

**Authors:** Ashley J Malin, Christine Till

**Affiliations:** Department of Psychology, York University, Keele St., 4700 Toronto, Canada

**Keywords:** ADHD, Water fluoridation, Neurotoxicity, Environmental factors

## Abstract

**Background:**

Epidemiological and animal-based studies have suggested that prenatal and postnatal fluoride exposure has adverse effects on neurodevelopment. The aim of this study was to examine the relationship between exposure to fluoridated water and Attention-Deficit Hyperactivity Disorder (ADHD) prevalence among children and adolescents in the United States.

**Methods:**

Data on ADHD prevalence among 4-17 year olds collected in 2003, 2007 and 2011 as part of the National Survey of Children’s Health, and state water fluoridation prevalence from the Centers for Disease Control and Prevention (CDC) collected between 1992 and 2008 were utilized.

**Results:**

State prevalence of artificial water fluoridation in 1992 significantly positively predicted state prevalence of ADHD in 2003, 2007 and 2011, even after controlling for socioeconomic status. A multivariate regression analysis showed that after socioeconomic status was controlled each 1% increase in artificial fluoridation prevalence in 1992 was associated with approximately 67,000 to 131,000 additional ADHD diagnoses from 2003 to 2011. Overall state water fluoridation prevalence (not distinguishing between fluoridation types) was also significantly positively correlated with state prevalence of ADHD for all but one year examined.

**Conclusions:**

Parents reported higher rates of medically-diagnosed ADHD in their children in states in which a greater proportion of people receive fluoridated water from public water supplies. The relationship between fluoride exposure and ADHD warrants future study.

**Electronic supplementary material:**

The online version of this article (doi:10.1186/s12940-015-0003-1) contains supplementary material, which is available to authorized users.

## Background

Attention-Deficit Hyperactivity Disorder (ADHD) is the most common neurodevelopmental disorder of childhood [1]. It is characterized by symptoms of inattention, impulsivity/hyperactivity or both that are present in childhood and can persist into adulthood [2]. As of 2011, 11% of 4-17 year olds in the United States (U.S.) had received a diagnosis at some point in their lives [3]. The high prevalence of ADHD is a growing public health concern because the behavioural symptoms of the disorder can seriously affect learning and academic achievement, as well as social functioning.

ADHD is considered to develop from an interaction between genetic and environmental factors [4-6], with numerous developmental neurotoxicants significantly increasing the risk for a diagnosis of ADHD. Environmental factors include prenatal and neonatal exposure to manganese [7], poly-chlorinated biphenyls (PCBs) [8,9], nicotine [10] and mercury [11,12], as well as childhood exposure to arsenic [13,14], food additives and food colouring [15], pesticides [16] and lead [17]. Fluoride however, despite being environmentally widespread and having demonstrable developmental neurotoxic effects, at a sufficient dose [18,19], has received virtually no attention in the ADHD literature. Nevertheless, there is a burgeoning body of human and animal research indirectly suggesting that it may contribute to the disorder’s onset.

### Water fluoridation and ADHD

The U.S. is one of the most widely fluoridated countries in the world, with approximately 74.6% of the population on public water systems and 67.1% of the total population receiving fluoridated water from public water systems for the prevention of dental caries [20]. The vast majority of those on fluoridated public water systems receive fluoride via the addition of fluoridation chemicals, while a small minority receives naturally occurring fluoride. Fluoridation chemicals include: hydrofluorosicilic acid, sodium fluorosilicate and sodium fluoride [21]. Until September 2010, the CDC’s Department of Health and Human Services (DHHS) recommended that U.S. public water systems be fluoridated at 0.7 – 1.2 mg/L [22]; however, they have found that children and adults living in communities fluoridated at this range actually tend to receive 0.9 - 3.6 mg and 0.6 - 6.6 mg per day respectively from all sources, including: water, food and dental products [23]. Consistently, the 2010 U.S. National Health and Nutrition Survey found that approximately 41% of 12-15 year olds suffer from dental fluorosis, a consequence of fluoride overexposure [24]. The DHHS has since announced a proposal to change the recommended fluoride concentration to 0.7 mg/L, but this has yet to be widely adopted [22,25].

Rats exposed to fluoridation chemicals have been shown to exhibit ADHD-like symptoms. Male rats whose mothers were injected with 0.13 mg/L of sodium fluoride two to three times per day during gestation days 14-18 or 17-19 had symptoms of hyperactivity at nine weeks of age. Juvenile and adult rats who drank water fluoridated at 100 mg/L for six weeks and 125 mg/L for 11 weeks, respectively, exhibited hypoactivity and impaired attention [26]. Although postnatal drinking water fluoride concentrations were high, blood plasma levels ranged from 0.059 - 0.640 mg/L, and these are comparable to plasma levels in humans who ingested 5 – 10 mg/L of fluoride [26,27]. Moreover, impaired learning and memory have also been found among rats that drank 5 mg/L of sodium fluoride treated water for six months or 20 mg/L for three months [28,29].

Rats with fluorosis also tend to have significant decreases in neural nicotinic acetylcholine receptors (nACHRs) and inhibited cholinesterase expression [30-33], both of which could interfere with attentional processes [34]. Moreover, they have significant decreases in protein expression of *α*4 and α7 nAChR subunit genes [28,31,35], and abnormalities at the *α*4 nAChR subunit in particular have been implicated in all ADHD subtypes [36,37]. Furthermore, nicotinic receptor agonists that ameliorate ADHD symptoms do so in rats by acting on the *α*_4_*β*_2_ and, in some cases, α_7_ subunits [38-41].

Fluoride can readily cross the placenta, accumulate in the infant brain and easily exert neurotoxic effects, such as decreasing norepinephrine in the parietal and occipital lobes, decreasing serotonin in the parietal lobe and increasing serotonin in the frontal and occipital lobes [42-45]. Such changes can adversely affect arousal and attention, pain tolerance, and learning and memory respectively [42,43]. Expectedly, prenatal fluoride exposure has been associated with impaired infant neurobehavioural development. For example, infants whose mothers lived in areas with water fluoridated at 1.7 to 6 mg/L while pregnant had delayed orientation reactions when compared to those whose mothers were exposed to 0.5 to 1.0 mg/L [46].

Exposure to fluoridated water during childhood has also been associated with impaired attention and cognitive and intellectual functioning. Importantly, among children who were exposed environmentally to water fluoridated at 1.2 - 3 mg/L (slightly above the U.S. recommended level), increased urinary fluoride concentrations were associated with slower reaction time and poorer visuospatial organization that could interfere with attention, and reading and writing respectively [47]. Additionally, urinary fluoride of 5.6 ± 1.7 mg/L was inversely related to performance on a measure of visual memory and visuospatial organization, as well as attention (the Rey-Osterrieth Complex Figure Test) [48]. A recent meta-analysis, which included a number of epidemiological studies, also found that children living in ‘high fluoride’ areas had IQs that averaged 7 points lower than those living in ‘low fluoride’ areas [49]. Seven of the ‘high fluoride’ areas had fluoride concentrations slightly above the U.S recommended range (1.8 - 3 mg/L) [50-56], while one had a concentration within the recommended range (0.88 mg/L) [57]. Moreover, a dose–response relationship between exposure to water fluoridated at relatively low concentrations (0.24 - 2.84 mg/L) and reduced IQ among children has also been established [58]. The association between fluoride exposure and lowered IQ in children provides support for a neurotoxic developmental effect. While ADHD was not measured in these epidemiological studies, it is plausible that fluoride is also contributing to attention-related symptoms given its association with lower IQ.

Using an ecological design, the current study examined whether higher water fluoridation prevalence is associated with higher rates of ADHD diagnoses in the U.S.. Given the research linking exposure to fluoridated water to adverse neurodevelopmental and cognitive effects, it was hypothesized that states with more widespread water fluoridation would tend to have higher ADHD prevalence.

## Methods

### ADHD sample

State-based ADHD prevalence data was obtained from the Centers for Disease Control and Prevention (CDC) website. The CDC collected this information via the National Survey of Children’s Health (NSCH). The NSCH is a cross-sectional random-digit survey, conducted in 2003, 2007 and 2011, in which parents were contacted via telephone and asked about the emotional and physical well-being of a randomly selected child from their household. To determine ADHD prevalence, each responding parent or guardian was asked whether “a doctor or other health care provider ever told you that [child] had attention deficit disorder or attention-deficit/hyperactivity disorder, that is, ADD or ADHD”. In the 2007 and 2011 NSCH, if the parent answered yes, he or she was asked whether the child was currently diagnosed with ADHD and, if so, how severe it is. In 2011, the responding parent was also asked the age of diagnosis [59]. Lifetime parent-reported health care provider-diagnosed ADHD (whether a parent or guardian had ever been told by a health care provider that his or her child had ADD or ADHD) was the measure of ADHD prevalence used in this study.

Extracted from the original sample of children aged 0-17, three subsamples of children aged 4-17 living in the U.S. were used to assess ADHD prevalence per state in 2003 (n = 79,264), 2007 (n = 73,123), and 2011 (n = 76,015). The lifetime prevalence of ADHD increased over time and was 7.8% in 2003, 9.5% in 2007, and 11% in 2011. ADHD prevalence was also higher for males, children of lower socioeconomic status (SES), older children, and for children whose parents had a high school education as compared to those whose parents either did not graduate high school or attained postsecondary education [3].

### Water fluoridation prevalence data

Data on the number of people receiving fluoridated water from public water supplies in each of the 50 states and the District of Columbia in 1992 (n = 144,217,476), 2000 (n = 161,924,080), 2002 (n = 172, 209,735), 2004 (n = 180,632,481), 2006 (n = 184,028,038), and 2008 (n = 195,545,109) was also obtained from the CDC website [20]. To determine state-based fluoridation prevalence, the CDC obtained and analyzed data from the Water Fluoridation Reporting System (WFRS), an online tool monitoring the percentage of the U.S. population on public water systems that receives optimally fluoridated drinking water [20]. For the years 1992, 2006 and 2008 the CDC distinguished between the number of people in the U.S. receiving fluoridation chemicals versus naturally occurring fluoride. Additionally, for 1992 only, the CDC distinguished between the prevalence of artificially versus naturally fluoridated water per state. In 1992, approximately 93.4% of people on fluoridated public water systems received fluoridation chemicals, while 6.6% exclusively received naturally occurring fluoride. In both 2006 and 2008, approximately 95.5% received fluoridation chemicals and 4.5% received natural fluoride.

To calculate the percentage of each state receiving optimally fluoridated (i.e. according to the DHHS recommendations) water from public water systems (i.e. encompassing either naturally or artificially fluoridated water) state population estimates were obtained from the United States Census website [60,61]. The number of people receiving optimally fluoridated water in each state was divided by the number of people in each state for a given year and multiplied by 100. For 1992, the number of people receiving artificially fluoridated water and the number receiving naturally fluoridated water in each state were also divided by the state population estimate and multiplied by 100 to determine the respective state based prevalence.

### Statistical analysis

Descriptive statistics were calculated for U.S. water fluoridation prevalence for all years examined. Statistical comparisons of ADHD prevalence and water fluoridation prevalence between geographic regions were determined using one-way ANOVA followed by Bonferroni post hoc test in all cases except for regional fluoridation prevalence comparisons in 2000 and 2002. In those cases Games-Howell’s test was used due to heterogeneous variances. Pearson correlations were used to examine relationships between state water fluoridation prevalence and state ADHD prevalence. These were not corrected for family wise error given the exploratory nature of this study. Hierarchical and multivariate regression analyses were conducted to examine the relationship between artificial water fluoridation prevalence and ADHD prevalence after controlling for natural water fluoridation prevalence and SES, and SES respectively. A one-tailed alpha level of 0.05 was the criterion for statistical significance for all analyses. A Bonferroni correction was applied to the univariate analysis of the multivariate regression however, making the criterion for significance for that analysis an alpha of 0.017.

## Results

### State water fluoridation

Median percentages and interquartile ranges of the U.S. population receiving optimally fluoridated water from public water systems in 1992, 2000, 2002, 2004, 2006 and 2008 are presented in Table 1. Median water fluoridation prevalence ranged from 58.16 - 66.33% from 1992-2008, increasing over time. Interquartile ranges ranged from 26.99 - 31.83%, indicating that fluoridation prevalence between states was highly variable.

**Table 1:** **Percentage of each state receiving fluoridated water per year**

**Year**	**Median**	**Interquartile Range**
1992	58.16	30.33
2000	58.62	31.83
2002	63.93	29.61
2004	66.24	26.99
2006	65.75	30.52
2008	66.33	30.39

### ADHD and water fluoridation prevalence according to geographic region

ADHD and water fluoridation prevalence were organized in Tables 2 and 3 respectively according to the United States Census Bureau’s classification of geographic regions [62] (See Additional file 1). Differences in ADHD prevalence between geographic regions were statistically significant in 2003 (F (3, 47) = 21.84, p = .000), 2007 (F (3, 47) = 12.07, p = .000), and 2011(F (3, 47) = 13.35, p = .000). In 2003, ADHD prevalence was significantly lower in the West (M = 6.41, SD = 0.8) than in all other regions, and in both 2003 and 2007 significantly higher in the South (M = 9.41, SD = 1.05 and M = 11.74, SD = 2.28, respectively), than in all other regions. In 2007 and 2011, ADHD prevalence was lower in the West (M = 7.73, SD = 1.3 and M = 8.75, SD = 1.67, respectively) than in all other regions, but not significantly lower than the North East (M = 9.46, SD = 0.97 and M = 10.96, SD = 1.72, respectively). In 2011, ADHD prevalence was highest in the South (M = 13.51, SD = 2.49), but not significantly higher than the Midwest (M = 11.93, SD = 2.03).

**Table 2:** **Prevalence of ADHD as a function of geographic region**

	**2003**		**2007**		**2011**	
**Region**	**Mean %**	**SD**	**Mean %**	**SD**	**Mean %**	**SD**
Northeast	7.92	1.13	9.46	0.97	10.96	1.72
Midwest	7.87	1.05	9.82	2.03	11.93	2.03
South	9.41	1.05	11.74	2.28	13.51	2.49
West	6.41	0.80	7.73	1.28	8.75	1.67

Note. Mean percentage of children or adolescents ages 4–17 ever diagnosed with ADHD as of that year; SD, standard deviation. Northeast, n = 9, Midwest, n = 12.

South, n = 17, West, n = 13.

**Table 3:** **Prevalence of water fluoridation as a function of geographic region**

	**1992**		**2000**		**2002**		**2004**		**2006**		**2008**	
**Region**	**M**	**SD**	**M**	**SD**	**M**	**SD**	**M**	**SD**	**M**	**SD**	**M**	**SD**
Northeast	39.6	22.36	49.39	22.60	50.79	22.0	49.78	19.25	50.30	19.43	50.13	21.39
Midwest	69.1	11.84	69.62	9.32	72.51	10.77	73.25	10.69	72.87	10.78	70.17	13.11
South	69.0	15.11	67.80	16.17	71.68	14.74	74.82	15.11	74.37	15.85	73.37	17.51
West	31.7	22.78	34.13	20.70	37.26	20.86	39.90	19.5	41.16	19.35	43.65	19.78

Note. M, mean percentage of population receiving fluoridated water from public water systems in that year. SD, standard deviation. Northeast, n = 9, Midwest, n = 12, South, n = 17, West, n = 13.

Differences in water fluoridation prevalence between regions were also statistically significant in 1992 (F (3, 47) = 15.05, p = .000), 2000 (F (3, 47) = 12.21, p = .000), 2002 (F (3, 47) = 13.20, p = .000), 2004 (F (3, 47) =15.07, p = .000), 2006 (F (3, 47) = 13.28, p = .000), and 2008 ( F (3, 47) = 8.88, p = .000). Similar to ADHD prevalence, water fluoridation prevalence in all years examined was lower in the West than in all other regions, but not significantly lower than the North East. In 2004, 2006 and 2008 water fluoridation prevalence was also higher in the South than in all regions, but not significantly higher than the Mid-West.

### The relationship between ADHD prevalence and water fluoridation prevalence

Since artificial and natural water fluoridation prevalence per state was only distinguished in 1992, the relationship between each and ADHD prevalence was of primary focus and examined separately. States with higher artificial fluoridation prevalence had significantly higher ADHD prevalence in 2003 (r (49) = .46, p = .000), 2007 (r (49) = .42, p = .001), and 2011 (r (49) = .48, p = .000). Natural fluoridation prevalence in 1992 however, was not significantly related to ADHD prevalence in 2007 or 2011, r (49) = −.19, p = .09, and r (49) = −.22, p = .06 respectively, but was significantly negatively associated with ADHD prevalence in 2003, r (49) = −.29, p = 0.02.

The relationship between overall state water fluoridation prevalence (not differentiating between artificial and natural fluoridation) and state ADHD prevalence in later years was also examined. Positive associations were found between the two for all years examined, except between water fluoridation prevalence in 2008 and ADHD prevalence in 2007 (p = .07). These correlations were numerically smaller however, than between artificial water fluoridation prevalence and ADHD prevalence (see Table 4).

**Table 4:** **Pearson correlations among water fluoridation prevalence and ADHD prevalence**

**Variables**	**2**	**3**	**4**	**5**	**6**	**7**	**8**	**9**
1.) ADHD2003	.67	.65	.32*	.37**	.38**	.39**	.39**	.32*
2) ADHD2007	―	.71	.35**	.30*	.30*	.31*	.28*	.21
3.) ADHD2011	―	―	.39**	.34**	.32*	.34**	.33**	.25*
4.) FPrev_1992	―	―	―	.82	.80	.81	.80	.75
5.) FPrev_2000	―	―	―	―	.96	.91	.91	.89
6.) FPrev_2002	―	―	―	―	―	.96	.97	.93
7.) FPrev_2004	―	―	―	―	―	―	.99	.95
8.) FPrev_2006	―	―	―	―	―	―	―	.96
9.) FPrev_2008	―	―	―	―	―	―	―	―

Note. ADHD, parent-reported health care provider-diagnosed lifetime prevalence of ADHD in that year. FPrev_, percentage of the population receiving fluoridated water from public water systems in that year. *p < .05, **p < .01. When not corrected for family-wise error, simple Pearson r > .25 is significant at p = .05, r > .33 is significant at p = .01.

### ADHD prevalence, SES and artificial water fluoridation prevalence

Those of lower SES are often targets of public artificial water fluoridation programs [63,64] and also tend to have higher ADHD prevalence [3]. Therefore, data on median household income per state in 1992 was obtained from the U.S. Census website [65] to examine whether SES could be mediating the relationship between artificial water fluoridation prevalence and ADHD prevalence. States with lower median household income in 1992 had significantly higher artificial water fluoridation prevalence in 1992 (r (49) = −.27, p = 0.03) and consistent with the NSCH findings, significantly higher ADHD prevalence in 2003 (r (49) = −.35, p = .006), 2007 (r (49) = −.37, p =. 007) and 2011 (r (49) = − .44, p = 0.001). Therefore, a hierarchical regression analysis was conducted to examine whether higher artificial water fluoridation prevalence in 1992 predicted higher prevalence of ADHD in 2003 after controlling for natural water fluoridation prevalence and median household income in 1992. These results are presented in Table 5.

**Table 5:** **Hierarchical regression predicting 2003 ADHD prevalence with 1992 artificial and natural fluoridation prevalence**

**Variables**	**Total R** ^**2**^	**∆ R** ^**2**^	**F change**	**df**	**B**
Step 1	.21	.21	13.11**	1, 49	
ArtF_1992					.027**
Step 2	.24	.03	1.75	1, 48	
ArtF_1992					.024**
NatF_1992					-.043
Step 3					
ArtF_1992	.34	.10	6.87*	1, 47	.017*
NatF_1992					-.071*
SES_1992					-.010**

Note. ArtF, prevalence of artificial water fluoridation. NatF, Prevalence of natural water fluoridation.

SES, median household income. B, unstandardized coefficient.

*p < .05, **p ≤ .01.

The final model was significant, F (3, 47) = 7.91, p = 0.000, and accounted for 33.5% of the variance in 2003 parent-reported health care provider-diagnosed ADHD. In the final model, artificial water fluoridation prevalence significantly and independently positively predicted 2003 ADHD prevalence, B = 0.017, t (47) = 2.16, p = 0.036, while natural water fluoridation prevalence and median household income in 1992 (measured in hundreds of dollars) significantly negatively predicted it, B = −0.071, t (47) = −2.21, p = 0.032 and B = − 0.010, t (47) = 2.62, p = 0.012 respectively. Therefore, while higher artificial fluoridation prevalence in 1992 was associated with higher parent-reported health care provider-diagnosed ADHD prevalence in 2003, higher natural fluoridation prevalence and median household income in 1992 were each associated with lower 2003 ADHD prevalence.

A multivariate hierarchical regression analysis was also conducted to examine the unique relationships between artificial fluoridation prevalence and ADHD in all three years of interest after median household income in 1992 was controlled (see Table 6). Natural water fluoridation prevalence in 1992 was not included in this model because it was not significantly correlated with ADHD prevalence in 2007 or 2011, and was already controlled for in the previous regression predicting 2003 ADHD prevalence.

**Table 6:** **Multivariate regression predicting ADHD prevalence with 1992 artificial fluoridation prevalence and 1992 median household income**

**Variables**	**B**	**SE**	**t**	**p value**	**[95% CI]**
ADHD 2003					
ArtF_1992	.023	.008	3.05	.004	.008, .038
SES_1992	-.007	.004	−1.92	.061	-.015, .000
ADHD 2007					
ArtF_1992	.031	.012	2.64	.011	.007, .055
SES_1992	-.013	.006	−2.17	.035	-.025, −.001
ADHD 2011					
ArtF_1992	.042	.013	3.20	.002	.015, .068
SES_1992	-.018	.007	−2.77	.008	-.031, −.005

Note. ArtF, prevalence of artificial water fluoridation. SES, median household income. ADHD. Parent-reported health care provider-diagnosed lifetime prevalence of ADHD, in the given year. B, unstandardized coefficient. Bonferroni corrected criterion for statistical significance, p < 0.017.

**Figure 1:**
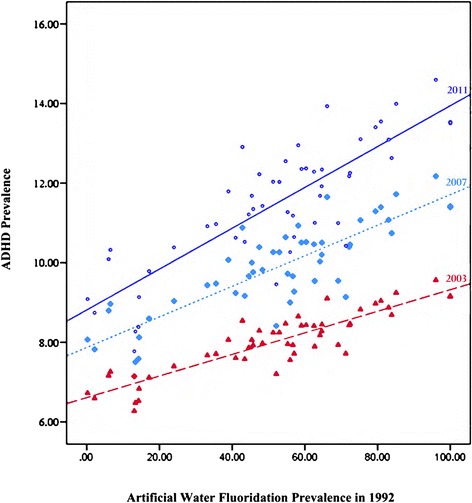
**Artificial fluoridation prevalence predicting ADHD prevalence after adjusting for 1992 median household income, by state.** The line with large dashes and triangles represent predicted values of ADHD prevalence in 2003. The line with small dashes and diamonds represent predicted values of ADHD prevalence in 2007. The solid line and circles represent predicted values of ADHD prevalence in 2011.

The overall model was significant when predicting ADHD prevalence in 2003 (F (2, 48) = 8.71, p = 0.001), 2007 (F (2, 48) = 7.94, p = 0.001) and 2011 (F (2, 48) = 12.21, p = 0.000), accounting for 24%, 22% and 31% of the variance in ADHD prevalence respectively. In the final model, artificial fluoridation prevalence in 1992 significantly and independently predicted parent-reported health care provider-diagnosed ADHD in all three years examined, Wilks λ = .81, F (3, 46) = 3.64, p = 0.02, while the predictive relationship between median household income in 1992 and ADHD prevalence in all three years was reduced to that of a trend, Wilks λ = .86, F (3, 46) = 2.48, p = 0.07. After applying a Bonferroni correction, artificial fluoridation prevalence in 1992 significantly predicted ADHD prevalence in 2003, (B = 0.023, t (48) = 3.05, p = 0.004), 2007 (B = 0.031, t (48) = 2.64, p = 0.011), and 2011 (B = 0.042, t (48) = 3.20, p = 0.002). Thus, after adjusting for socioeconomic status, a 1% increase in artificial water fluoridation prevalence in 1992 was associated with a 0.023% increase in ADHD prevalence in 2003 (corresponding to approximately 67,000 additional diagnoses), a 0.031% increase in ADHD prevalence in 2007 (corresponding to approximately 93,000 additional diagnoses) and a 0.043% increase in ADHD prevalence in 2011 (corresponding to approximately 131,000 additional diagnoses). Median household income in 1992 (measured in hundreds of dollars) did not meet the threshold for significance in predicting ADHD prevalence in 2003 (p = 0.061) or 2007 (p = 0.035), but did so in 2011 (B = −.018, t = − 2.77, p = 0.008) (see Figure 1).

## Discussion

Fluoride has been shown to have developmental neurotoxic effects [18,19] and to be associated with impaired cognitive functioning in infants and children. This is the first study to examine the relationship between exposure to fluoridated water and ADHD prevalence, and did so using population-based data collected by the CDC. It is also unique in that it examined ADHD prevalence within the U.S., decreasing the likelihood that differences in ADHD prevalence between states reflect differing diagnostic criteria (DSM criteria is most commonly applied in the U.S. to diagnose ADHD). Furthermore, ADHD state prevalence was determined using identical methodology, eliminating the common problem of differing methodologies when comparing ADHD prevalence between countries [1].

As hypothesized, water fluoridation prevalence was positively associated with parent-reported health care provider-diagnosed ADHD prevalence. Geographic regions and states in which a greater proportion of people received fluoridated water from public water systems tended to have a greater proportion of children and adolescents diagnosed with ADHD. This suggests that living in an “optimally” fluoridated community increases a child or adolescent’s risk of developing the disorder. Moreover, results did not appear to be confounded by socioeconomic status because they remained consistent after controlling for this variable. Our findings are consistent with prior epidemiological studies that have associated high and low fluoride concentration exposure [49,58] with neurodevelopmental effects in children.

Artificial water fluoridation prevalence was significantly positively associated with ADHD prevalence, while natural water fluoridation prevalence was either negatively or not significantly associated with it. Although this could imply that the relationship between exposure to fluoridated water and increased ADHD prevalence is specific to fluoridation chemicals, the high variability in naturally occurring fluoride concentrations (0.1 mg/L - 15.9 mg/L) [21] within states prevents this conclusion from being made. Specifically, natural fluoride concentration could potentially be confounding the relationship between natural fluoridation prevalence and ADHD prevalence leading to a misleading result. For example, counties with low natural fluoridation prevalence could have high concentrations of naturally occurring fluoride that pose a greater neurodevelopmental risk than high prevalence of low concentrations of naturally occurring fluoride. This could contribute to increased ADHD prevalence within states that have low natural fluoridation prevalence. Thus, future research controlling for the high variability in natural fluoride concentration is necessary to more validly examine this relationship. Additionally, unlike artificially fluoridated water, U.S. citizens can be exposed to naturally fluoridated water from sources other than public water systems (e.g. wells and springs). Therefore, the state prevalence of natural fluoridation from public water systems may not reflect the true state-based proportion of people exposed to naturally fluoridated water.

Since states of lower SES tended to have higher artificial water fluoridation prevalence and ADHD prevalence, another important area of investigation was whether artificial water fluoridation prevalence in 1992 still predicted ADHD prevalence after SES was considered. That is, did children and adolescents in states with higher artificial water fluoridation prevalence merely have higher rates of ADHD because they tended to be of lower socioeconomic status and therefore more likely to have additional ADHD risk factors? Results showed that this was not the case and prevalence of artificial water fluoridation in 1992 did indeed predict ADHD prevalence independent of SES. Moreover, artificial water fluoridation prevalence even appeared to be the more robust predictor.

Although more research is needed to investigate the relationship between exposure to fluoridated water and increased ADHD prevalence, there are two main pathways by which exposure to fluoridated water could theoretically contribute to the disorder. First, silicofluoride-treated water has been shown to corrode lead-bearing plumbing, increasing the leaching of lead in the water [66]. Silicofluorides appear to react synergistically with lead, which in turn, increases its uptake into the body [27]. Consequently, children living in communities with silicofluoride-treated water tend to have increased lead venous blood levels (VBLs) (above 5 μg/dL), and those with additional risk factors for lead exposure (e.g. living in a house built before 1939 or living in poverty during the ages of 0-5) appear most vulnerable [67-70]. Lead VBLs equal to and lower than those more commonly found among children living in silicofluoride-treated communities have repeatedly been associated with a significantly increased risk of developing ADHD [15,71]. In fact, it has been suggested that 25.4% (598 000) of ADHD cases among 8-15 year olds in the U.S. could be attributed to lead exposure greater than 1.3 μg/dL [72].

Second, exposure to fluoridated water may contribute to ADHD via suppression of the thyroid gland. Fluoride reduces thyroid gland activity [73-75] and thyroid hormones are particularly important for cholinergic activity in the basal forebrain and hippocampus [76]. Moreover, hypothyroxemia has been associated with ADHD and is considered a potential cause of the disorder [77]. In fact, thyroid gland suppression is the mechanism by which PCB exposure contributes to it [78]. Additional studies are necessary to investigate the interaction among fluoride exposure, thyroid function and ADHD symptoms and to clarify whether exposure to fluoridated water contributes to ADHD via suppression of the thyroid gland.

Even though current findings indicate a relationship between ADHD prevalence and fluoride exposure that occurs through the optimal fluoridation of public water systems, there are several study design limitations that should be considered. First, this study is an ecological design that broadly categorized fluoride exposure as exposed versus non-exposed rather than collecting information related to concentration of fluoride and patterns and frequency of exposure or outcome at the individual level. Future research could explore the relationship between exposure to fluoridated water and the occurrence of ADHD at the individual level. Further clarification of a potential dose–response relationship between fluoride exposure and ADHD symptoms would also be important for determining causality. Second, given that fluoridation prevalence in neighboring years was highly correlated from 2000 onward and unavailable for the mid to late 90s, it could not be determined whether exposure to fluoridated water at a particular period of development was most associated with increased ADHD prevalence. Nevertheless, given that other research has demonstrated the developing brain’s particular sensitivity to the neurotoxic effects of fluoride, it is likely that prenatal and early postnatal development presents a window of vulnerability. Third, fluoridation prevalence data was analyzed with ADHD prevalence data from different years, and therefore, it cannot be confirmed that those surveyed in a given year were living in the same region as when the fluoridation data were derived. Fourth, we were unable to obtain reliable population-based data on blood lead levels among 4-17 year old children and adolescents, and therefore could not determine whether lead was mediating the relationship between exposure to fluoridated water and ADHD. Lastly, parent-reported health-care provider-diagnosed ADHD prevalence was used in this study which is not as precise a measure as others (e.g. conducting formal ADHD assessments) or may be subject to potential parent biases regarding seeking or accepting an ADHD diagnosis for their child. Therefore, the survey method used in the current study may not completely capture ‘true’ ADHD prevalence. Despite these limitations, an association between exposure to fluoridated water and ADHD prevalence was still found, even after considering the increased tendency for children in low SES states to receive an ADHD diagnosis.

## Conclusions

In summary, this study has empirically demonstrated an association between more widespread exposure to fluoridated water and increased ADHD prevalence in U.S. children and adolescents, even after controlling for SES. The findings suggest that fluoridated water may be an environmental risk factor for ADHD. Population studies designed to examine possible mechanisms, patterns and levels of exposure, covariates and moderators of this relationship are warranted.

## Additional file

Additional file 1:
**US Census Regions and Divisions.** Census regions and divisions of the United States. This file includes a map of the U.S. census regions and divisions as well as a list of the states in each region and division.
