# Supplementary material for: Exposure to fluoridated water and attention deficit hyperactivity disorder prevalence among children and adolescents in the United States: an ecological association
Source: Environ Health. 2015 Feb 27;14:17. doi: 10.1186/s12940-015-0003-1 (PMC4389999; doi:10.1186/s12940-015-0003-1)
Supplement: Additional file 1: — US Census Regions and Divisions. Census regions and divisions of the United States. This file includes a map of the U.S. census regions and divisions as well as a list of the states in each region and division. [file 12940_2015_3_MOESM1_ESM.pdf]

Census Regions and Divisions of the United States

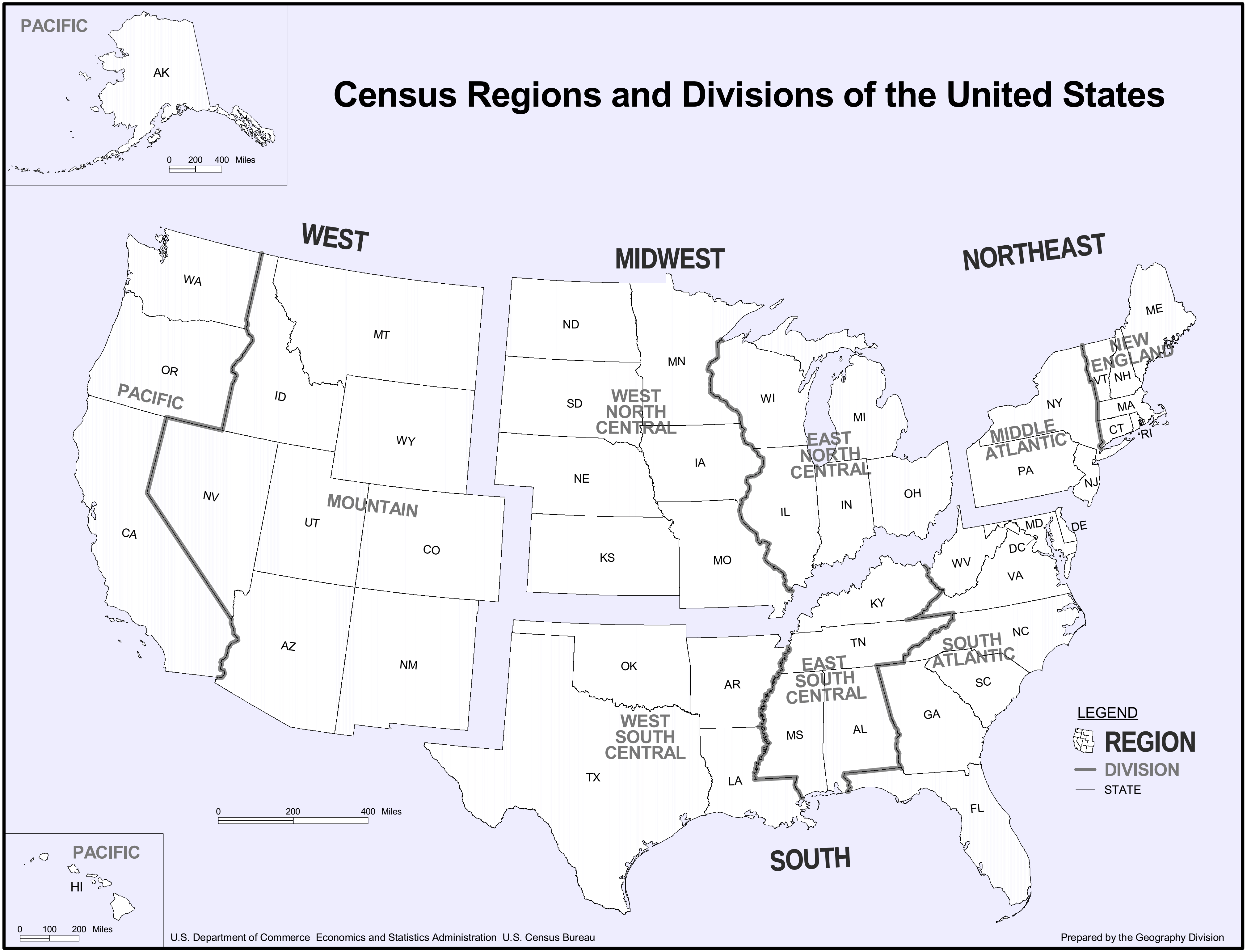

U.S. Department of Commerce Economics and Statistics Administration U.S. Census Bureau

Prepared by the Geography Division

U.S. Census Bureau

Census Bureau Regions and Divisions with State FIPS Codes

Region I: Northeast

Division I: New England

Connecticut (09)
Maine (23)
Massachusetts (25)
New Hampshire (33)
Rhode Island (44)
Vermont (50)

Division 2: Middle Atlantic

New Jersey (34)
New York (36)
Pennsylvania (42)

Region 2: Midwest*

Division 3: East North Central

Indiana (18)
Illinois (17)
Michigan (26)
Ohio (39)
Wisconsin (55)

Division 4: West North Central

Iowa (19)
Kansas (20)
Minnesota (27)
Missouri (29)
Nebraska (31)
North Dakota (38)
South Dakota (46)

Region 3: South

Division 5: South Atlantic

Delaware (10)
District of Columbia (11)
Florida (12)
Georgia (13)
Maryland (24)
North Carolina (37)
South Carolina (45)
Virginia (51)
West Virginia (54)

Division 6: East South Central

Alabama (01)
Kentucky (21)
Mississippi (28)
Tennessee (47)

Division 7: West South Central

Arkansas (05)
Louisiana (22)
Oklahoma (40)
Texas (48)

Region 4: West

Division 8: Mountain

Arizona (04)
Colorado (08)
Idaho (16)
New Mexico (35)
Montana (30)
Utah (49)
Nevada (32)
Wyoming (56)

Division 9: Pacific

Alaska (02)
California (06)
Hawaii (15)
Oregon (41)
Washington (53)

**Prior to June 1984, the Midwest Region was designated as the North Central Region.*
